# Experience of pediatric nurses in nursing dying children - a qualitative study

**DOI:** 10.1186/s12912-023-01274-0

**Published:** 2023-04-18

**Authors:** Weina Bian, Junxiang Cheng, Yue Dong, Ying Xue, Qian Zhang, Qinghua Zheng, Rui Song, Hongwei Yang

**Affiliations:** 1 Department of Nursing, Hanzhong Central Hospital, Shaanxi, 723000 China; 2 Department of Psychiatry, the First Hospital of Shanxi Medical University, Shanxi, 030000 China; 3 Department of ICU, the Affiliated Lianyungang Hospital of Xuzhou Medical University, Jiangsu, 222000 China; 4 Department of Statistics, Hanzhong Central Hospital, Shaanxi, 723000 China

**Keywords:** Nursing, Dying children, Experience, Qualitative research

## Abstract

**Purpose:**

To explore pediatric nurses’ challenges and effective coping strategies in caring for dying children.

**Methods:**

A descriptive qualitative study was adopted. Data were collected using a semi-structured interview with ten nurses from the pediatric, pediatric emergency, and neonatology departments.

**Results:**

Three themes were generated: stressors, consequences, and coping strategies. Ten sub-themes were generalized: negative emotions; helplessness; questioning rescue behavior; fear of communication; lack of workforce for night rescue; compassion fatigue; burnout; changes in life attitudes; self-regulation; leadership approval and no accountability.

**Conclusions:**

Through qualitative research, nurses’ challenges and effective coping strategies in caring for dying children were found, which provides information for nurses’ career development and related policy formulation in China.

**Clinical relevance:**

While there are many articles in China on hospice care, there is little research on the nurses’ experience of caring for dying children. Many studies have mentioned the adverse consequences of caring for dying children in foreign countries, leading to post-traumatic stress disorder (PTSD). However, domestic discussion of such problems is rare, and no corresponding coping strategies exist. This study explores pediatric nurses’ challenges and effective coping strategies in caring for dying children.

**Supplementary Information:**

The online version contains supplementary material available at 10.1186/s12912-023-01274-0.

## What is already known about this topic?

The experience of caring for dying children causes stress on nurses, which can affect their work and life, and even lead to post-traumatic stress disorder (PTSD). According to the studies that have already been published, this stress is mainly the feeling of helplessness and depression in facing the death of children, communication challenges, insufficient staff, and heavy workload. In coping strategies, there are two main ways of problem-centered coping and emotion-centered coping. Emotional support can reduce the incidence rate of post-traumatic stress disorder.

## What does this paper add?

This study finds that the stressor in caring for dying children differs in China. The first is negative emotions, such as sadness, dismay, and anxiety, especially in caring for children with long nursing hours. The other is questioning the rescue behavior, making nurses constantly recall the rescue process. Leadership approval and no accountability are also essential in coping strategies, except self-regulation of coping strategies in China.

## What are the implications of this paper?

The study guides nurses in China, especially nursing managers, to pay attention to the feelings of nurses who care for dying children. Moreover, it also provides some information for the development of nurses and the formulation of relevant policies.

## Introduction

Discussion of death is taboo in some countries. Parents control their emotions as much as possible when they learn their child could die [1]. There are few studies about the nurse’s role and experience in this case in China. Searching for this theme in WANFANG MED ONLINE in the past five years, one study was found about the effect of comprehensive nursing mode on children’s pain and psychological state in an intensive care unit (PICU) [2]. However, many studies have explored the experience of nurses in other countries in caring for dying children. According to the study, pediatric nurses faced significant challenges caring for children on the verge of death [3]. The sadness and pain resulting from a child’s death differ from an adult’s. According to a study published in 2019 by Hagen, Ter-Viola, and Graves, pediatric nurses are prone to negative workplace symptoms, such as compassion fatigue, burnout, sadness, and post-traumatic stress disorder (PTSD), especially in care for dying children [4]. To look up literature related to the care of children at the near-death stage, the authors used synonyms and keywords in the Academic Search Complete “、” CINAHL Complete “、” Health Source: Nursing /Academic Edition “、” MEDLINE “、” MEDLINE Complete” and other databases carried out a comprehensive search. Nurses’ challenges and coping strategies in caring for dying children were extracted. Search procedures include tracking critical databases using predefined terms relevant to the topic (see Appendix 1). Through filtering titles and abstracts through EBSCOhost software, 164 articles were extracted from 794 documents, excluding peer review, literature review, book chapters, meeting minutes, and other grey literature. All were published in English between 2010 and 2020 with the complete text. Finally, 20 articles were selected as references in this paper (see Appendix 2). Most of them ( n = 19) focused on the challenges and pressures nurses face in caring for such children, with more than half of the articles (n = 11) from the United States and the rest from Finland, Ireland, Colombia, Iran, Australia, Indonesia, Sudan, Canada, and South Korea.

First of all, with society’s development, people pay more and more attention to children’s health. Pediatric nurses face tremendous pressure, such as the rapid change in children’s conditions and the highly demanding nursing skills, especially at the critical moment of death [5]. In a qualitative study of seven nurses at a specialist hospital for children in Iran, it was found that nurses feel helpless and depressed in the face of the death of children they take care of, which may be related to the mutual attachment between nurses and children [6]. The second stressor is the communication challenge, which affects nurses, children, and families. Non-effective communication results in decline in family satisfaction [7–11]. In these studies, parents do not allow nurses to tell their children the truth. For example, in Hopia and Hino-Toronin’s study, one participant said she could not do what she thought was correct due to family demands [12]. Palliative care is best used for the rest of the child’s life to minimize the harm caused by over-treatment. However, the parents hold different views that if given enough treatment, the child could gradually recover [13]. —the most considerable communication challenge derived from children’s death. According to Kenan and Mike, after the child’s death, the contact between the nurse and the family was cut off, and the family did not want to have any contact with the nurse [14]. The third stressor is the insufficient staff and heavy workload [5].

Secondly is the impact of this care experience on nurses. In 20 studies, it was mainly reflected in three aspects: first is job burnout [15, 16]. In the study of 107 nurses in Indonesia, the author found that the long-term tense environment made nurses feel emotionally tired, thus lacking energy in their work. Unfriendly to patients and colleagues led nurses to give up their central [16]. The second is compassion fatigue, reported in six studies, which refers to emotional stress due to seeing much harm [17]. Negative situations can affect individual psychological changes, resulting in cold emotions. In the study of Berger et al., 239 American nurses were investigated. It found that day-to-day care of critically ill children induced nurses to develop sympathetic fatigue, which led to anxiety, depression, and other diseases of nurses. Because of a lack of enthusiasm for work, nurses were 18% more likely to make mistakes [18]. These findings are consistent with Nieran et al. [19] and the studies of Hagen, Ter-Viola, and Graves [4]. The third is post-traumatic stress disorder [5, 13, 16, 18–20]. These consequences seriously affect the work and life of nurses, leading to care. The efficiency of taxi work decreased, the turnover rate increased, and the quality of life decreased.

In coping strategies, there are two main ways of problem-centered coping and emotion-centered coping [3]. However, most nurses tend to be emotion-centered coping, consistent with Kellogg et al. [20]. studies have shown that nurses are emotionally stressed when caring for high-risk patients. A survey of 334 nurses in the United States found that emotional support reduced their risk of secondary traumatic stress. The study suggests that managers should pay attention to the emotional needs of nurses [7, 8, 12, 17]. Besides, a communication tool for families and caregivers is mentioned. [12]. This tool’s application ensures that nurses communicate confidently with the child’s family during or after the child’s death while providing a profound and meaningful experience for the family [12]. Therefore, some studies suggest that nursing managers focus on developing nurses’ coping skills in caring for dying children and provide necessary communication skills and professional knowledge training [21–23].

There are many articles on professional stress and coping strategies of nurses in China, such as mindfulness decompression therapy [24], employee assistance program service [25], and group psychological counseling based on focus solution technology [26]. However, there are few references to care for dying children. The author also experienced critical children’s death in 15 years of pediatric nursing and even caused severe traumatic stress, leading to insomnia, anxiety, and despair. Therefore, the author hopes to explore Chinese nurses’ main challenges in caring for dying children and determine whether effective coping strategies can help them overcome these challenges.

## Methods

### Design

There were five reasons for choosing a qualitative descriptive(QD)for this study. First, researchers generally draw from a naturalistic perspective and examine a phenomenon in its natural state [27]. Second, QD has been described as less theory-driven than other qualitative approaches [28], facilitating flexibility in commitment to a theory or framework when designing and conducting a study [27, 29]. For example, researchers may or may not decide to begin with a theory of the targeted phenomenon and need not stay committed to a theory or framework if their investigations take them down another path [29]. Third, data collection strategies typically involve individual and/or focus group interviews with minimally structured or semi-structured interview guides [27, 28]. Fourth, researchers commonly employ purposeful sampling techniques, such as maximum variation sampling, which has been described as helping obtain broad insights and rich information. Fifth, qualitative methods are used to understand better people’s thoughts, behaviors, and situations about certain phenomena [30]. As noted above, the results will likely read differently than those for a QD study [31]. Therefore, it is essential that researchers accurately label and justify their choices of approach, particularly for studies focused on participants’ experiences, which could be addressed with other qualitative traditions. Justifying one’s research epistemology, methodology, and methods allow readers to evaluate these choices for internal consistency, provides context to assist in understanding the findings, and contributes to the transparency of choices, enhancing the study’s rigor.

This study adopts the trustworthiness criteria of Lincoln and Guba (1994) [32, 33]. In order to ensure the validity and scientific accuracy of the data, the researchers collected data using live notes and audio recordings, while using maximum diversity sampling. Data were collected using a semi-structured interview with ten nurses from the pediatric, pediatric emergency, and neonatology departments. The content analysis method analyzes the obtained data, encodes sentence by sentence, and condenses the subject.

### Recruitment and sample

In this study, the participants were recruited by a poster in August 2021. In the recruitment poster, the author clarified the purpose of the study, the research method, and the inclusion and withdrawal criteria of the subjects. For two weeks, this poster was posted on the bulletin board of a Grade 3 A hospital in Hanzhong City, Shaanxi Province, and published in the WeChat group of nurses. Nurses who want to participate in the registration by email will receive an information questionnaire.

Inclusion criteria :(1) pediatric nurses have been working in hospitals for more than one year ;(2) being able to express their views clearly in Mandarin ;(3) obtaining informed consent ;(4) having the experience of caring for dying children in the past five years. The exclusion criteria were :(1) nurses who withdrew during the interview ;(2) nurses who did not want to mention their experience caring for dying children.

A total of 30 email replies were received. According to the inclusion and exclusion criteria, ten people from different departments were finally selected as the sample according to the study purpose. All of whom were female, aged 25–49, with an average age of 35.5, including six nurses with a college degree and four nurses with undergraduate degrees, all of whom were married and one divorced.

### Ethical considerations

In this study, the report will replace each participant with a code, and the interviewee’s identity, residence, and contact information will not be disclosed to others. Video Sound content is also used only in this study. After the study, the audio data will be destroyed. The hospital ethics committee approved this study.

### Data collection

Interested nursing staff requested to return to the questionnaire in October 2021, where the investigator contacted them, coded and negotiated the interview time, and signed informed consent before the interview. In November 2021, investigators used semi-structured interviews to collect data. The questions involved in the interviews are detailed in Appendix 4. The interview site is in a hospital’s family home dormitory for 20–40 min. The investigator will record the participant’s views and record them with the other party’s consent. The results will be returned to each participant in December to verify the interview details, thus ensuring the accuracy and credibility of the analysis. Before this interview, investigators were trained in interview and communication skills, including effective listening and giving positive feedback, establishing good relationships with interviewees, maintaining eye contact, not interrupting interviewees, not judging their views, etc. Besides, Kavanaugh and Ayers (1998) pointed out that in the course of the study, especially for sensitive topics, it is essential to assess participants’ feelings. Researchers must try to minimize the discomfort of participants. It is unethical if researchers cannot deal with pain [34]. Therefore, in this study, psychological counseling was prepared for participants to release their uneasy and uncomfortable feelings during or after the interview.

### Procedure and analysis

Content analysis by Krippendorff (2018) was used to analyze the experience of Chinese nurses caring for dying children. NVivo 12(QSR International) computer software is often used in qualitative research and can help with fast coding, deep exploration, and strict management:

Raw data is entered verbatim into the computer and then uploaded to the NVIVO.

The content is verbatim-coded. A concept label is then created for the nodes in the NVIVO, and the words under the concept are dragged to the nodes they support.

The content and topic are classified by application software.

## Results

This survey extracted three topics: stress sources, consequences, and coping strategies. See Appendix 5 for details.

### Stressors

Participants described the stress involved in dying children’s care, including negative emotions, feelings of helplessness, questioning their rescue behavior, communicating fear, and lack of workforce at night.

#### Negative emotions

Nine participants(NI-N9)mentioned that stress originated from adverse emotional reactions, including anxiety, sadness, frustration, crying, sadness, vomiting, hate, disgust, disappointment, guilt, complaints, dissatisfaction, discomfort, and fear. Some of the negative emotions were repeatedly mentioned by the respondents, such as the proportion of depression reached 66.67%, and crying and fear was 33.33%. One common manifestation of respondents with such negative emotions is that the more treatments are added to the child, the heavier the adverse emotional reaction will be.

Most participants mentioned their negative emotions, such as sadness, dismay, and anxiety, especially in caring for children with long nursing hours.

“Seeing my nursing child lying there with many tubes in her body, I felt so sad that I could not control my tears and wanted to cry,“ N1 said.

N6 said, “I even wanted him to die peacefully. Nevertheless, I felt very guilty because of this kind of thought… How can I have this thought? Even if I can not save him, I should try my best .“

N7 said, “I felt anxious because I do not want to do anything to increase his pain. I wanted to let him quietly walk the final course of life, but my responsibilities forced me to continue injecting medicine and intubation.“

#### Helplessness

Five respondents(N1,N2,N4,N7,N8) mentioned feelings of helplessness. Especially when the child eventually died, and the experience affected the respondents’ lives.

“I was afraid of hurting her, watching her look at me, watching her react get worse. I felt so bad I could not do anything.“ by N2.

N4 said, “I have done everything I can, but the kids are getting worse and worse. I felt useless.“

#### Questioning the rescue behavior

Four respondents(N1, N3, N5, N8) have questioned their rescue efforts. Two of them(N3, N5) lacked confidence in their nursing skills, and two(N1, N8) had higher requirements for their abilities and felt they could do better.

“Especially when the child’s condition suddenly changed, we rescued him together, but after the rescue, I would constantly recall the whole rescue process, was I wrong, where I operated slowly, what did I ignore?

#### Fear of communication

Three respondents(N6, N8, N9) mentioned a lack of communication skills and a fear of communicating with their families. Such respondents were questioned by their family members, and the family member was not present when the child’s condition deteriorated.

N8 said, “I dared not communicate with the child’s family. I fear telling them information that may differ from what the doctor said. I fear family emotional control, which would produce extreme behavior.“

“I wanted the mother to stop crying, but I did not know how to communicate with her because if I were her, I might cry worse than she did,“ N9 said.

#### Lack of workforce for night rescue

Four respondents(N2, N4, N7, N10) talked about a labor shortage at night, especially at night, without the support of mobile nurses.

N4 said, “I was most afraid of the night rescue because of the staff shortage. Other children need care. It was easy to make nursing errors.“

“If I had a child who would get worse at any time during the night shift, I would have been restless all night. I remembered one time, one child was being rescued, the other child was getting worse, and the other children’s families were calling me, and I cried as I rescued, wishing I had three heads and six arms.“ by N7.

### Stress consequences

This experience’s consequences include compassion fatigue, burnout, and changes in life attitudes.

#### Compassion fatigue

Four respondents(N1, N4, N6, N10) talked about the consequences of stress as compassion fatigue. Their common point is that they had experienced countless rescue efforts for the child, with N4 working less than ten years and the other three years working more than 20 years.

N4 said, “I felt that care for dying children is part of the job, without any feeling, just routine care and rescue.“

“I have saved too many children, no feeling. This is the life.“ by N6.

“When I was at work, I thought these kids were pathetic, but now, I did not feel that way at all,“ said N10.

#### Burnout

Five respondents(N3, N4, N6, N7, N10) talked about the consequences of stress, especially when they think of their children.

N4 said, “I have had many thoughts about leaving, especially every time I save a child, and I have had enough of this experience, which has seriously affected my mood. I did not want to be a pediatric nurse anymore. I have applied for a change with the leader but have not been approved.“

“If I had found a better job, I would have resigned, the pressure on the first-line pediatric nurse was too great, and I did not even want to go to work,“ said N7.

#### Changes in life attitudes

Six respondents(N1, N2, N3, N5, N8, N9) said that the consequences of stress were a change in life attitude and that the changes were all positive, making them more passionate about life and caring for their families.

N5 said, “This experience has greatly changed me, and life is fragile. I should love life more. I tried to care about my children and spent more time with my parents because of empathy. The parents of those children rushed to the hospital from their workplace and saw their children dying. The collapse of the look and the atonement deeply hurt me.“

“I have always felt I have much time to accompany my children, but accidents always happen. I did not care about that until I lost my child. So I could feel the feelings of the family very much. I went to study psychology and took a counselor, and I hope I can give the same experience as my parents a little help.“

### Coping strategies

Most nurses in this survey mentioned self-regulation, and individual nurses thought that if the head nurse or department director affirmed their behavior initially, not holding them accountable would significantly help them cope with stress.

#### Self-regulation

Ten respondents talked about self-mediation, releasing their pressure by watching music, watching TV, reading books, and slowly forgetting the experience of rescuing the children. In addition, respondents will not talk about their families, mainly afraid of burdening their families and affecting their emotions. Only two (N3, N5)mentioned finding friends or colleagues to communicate with.

“I was under much stress and wanted to be quiet after the rescue,“ said N2.

“It took a long time to adjust your negative emotions slowly each time.“ by N8.

N9 said, “It was all self-regulating, finding a quiet corner, not wanting to ask for help from others, who did not necessarily understand because they have not experienced it.“

#### Leadership approval, no accountability

Eight respondents(N1-N8) wanted support from a doctor or leader, especially support and encouragement from the head nurse. A hug and a word of approval can maximize the pressure.

N7 said, “Once I cried after rescuing the child, and I was agitated because the child was about the age of my daughter, and the head nurse came and hugged me, and she whispered to me,“ You were doing well.“

“What I fear most was leadership accountability, and I did not think I would be under pressure if the department director made it clear that the child’s death was not my responsibility.“ by N3.

## Limitations

Although this paper’s sample size is saturated, Chinese nurses do not talk much about the feelings of children dying, especially young nurses. The nurses who have worked for over ten years provide much information. This is different from other contentious. For example, in Ireland, nurses were invited to attend the children’s funerals [14]. However, Chinese nurses have almost no intersection with their families after the children’s death, so there is still a certain lack of research in digging deep into the psychological level of nurses. Further research recommends grading studies based on nurses’ working years, such as nurses who have been on the job for less than three years or more than ten years.

## Discussion

With the promotion of magnetic nursing in more and more hospitals in China, nurses’ physical and mental health is significant for developing high-quality holistic care [35]. This study found that the experience of pediatric nurses caring for dying children has some positive effects on their life and work, such as making nurses love life more and cherish the company of family. Some are negative, such as making nurses sympathetic fatigue and collapse.

There were ten subthemes in this study. Two aspects of stressors are different from foreign studies. One is that nurses question their rescue behavior. Because the investigation took place in a third-class hospital in Hanzhong City, there was no situation of foreigners seeking medical treatment, so there was no language barrier. Nurses questioning their rescue behavior mainly occurred when a child died unexpectedly. Because it happened so suddenly, the nurse repeatedly recalled what was wrong or missed in the whole process and whether a specific behavior caused the child’s condition to change. Studies have found that once a child dies suddenly, regardless of whether there is direct responsibility for the nurse, it will pressure the nurse psychologically. The other is various forms of negative emotions, especially the more treatment added to the child, the heavier the adverse emotional reaction of the nurse will be.

Regarding stress consequences, job burnout, and compassion fatigue are consistent with foreign research results [36–38], but nurses in this study do not mention post-traumatic stress disorder. It is worth noting that 60% of nurses mentioned that this experience had played a positive role in promoting the change in their life attitude, making them feel that life is so fragile that they should cherish life and love life more.

Different manifestations of coping strategies that most nurses in China tend to relieve stress through self-regulation, while in foreign studies, emotional support between teams is the mainstay [20]. Foreign results of coping with stress pay more attention to the mental health of nurses and the use of coping tools, such as the development and application of communication tools [12]. However, domestic nurses rarely mention the impact of external circumstances and characters on themselves. Only two people mentioned finding friends or colleagues to communicate with. However, 80% of the nurses mentioned their desire to get the doctor’s or leader’s support, especially the head nurse. The approval of the leader had an apparent effect on the pressure. Moreover, In this interview, nurses did not realize that external forces could be used to combat pressure, and nursing managers should have paid more attention to this emotional need of nurses.

## Conclusions

This study not only puts forward some guidance for nursing managers to focus on the feelings of nurses who care for dying children but also provides information for the career development of nurses and the formulation of relevant policies. Nursing managers should pay close attention to nurses caring for dying children, especially the first nurse, to give enough emotional support, let them the negative emotions, and coping strategies and related knowledge training, application of mature management tools to press the maximum to relieve their pressure.

## Electronic supplementary material

Below is the link to the electronic supplementary material.

Supplementary Material 1

Supplementary Material 2

## Data Availability

The data and materials used for analysis and conclusions are available from the corresponding author upon reasonable request.
