# Supplementary material for: Experience of pediatric nurses in nursing dying children - a qualitative study
Source: BMC Nurs. 2023 Apr 18;22:126. doi: 10.1186/s12912-023-01274-0 (PMC10111798; doi:10.1186/s12912-023-01274-0)
Supplement: Supplementary file 1 — Supplementary Material 1 [file 12912_2023_1274_MOESM1_ESM.docx]

**Interviewed person: N1**

1.What do you feel when you take care of dying children?

When I saw the child's body with a lot of tubes, and he was getting worse on the verge of death, I felt very sad, and I couldn't help crying.

2.What are your unique feelings about taking care of or rescuing such children?

I felt really sad to watch the life go by, especially when I didn't help the children.

3.How do you overcome or relieve this emotion?

I will try my best to do what I can do as much as possible, sometimes I forget that feeling.

4. Would you like to talk how to relieve this stress?

It often takes lots of time to ease, and only better after seeing the children's recovery and a positive response from their parents.

5.In this case, what do you think is the biggest challenge for you? How do you deal with this challenge? What ways or behaviors or people could help you overcome these challenges?

I think the biggest challenge I face is that the family can not accept the fact that the child was critically ill. They didn't believe me at all. They blamed my care and insulted me. I try to make sure I don't make mistakes and avoid bad accidents.A good security environment and the responsibility of my superiors will help me overcome the difficulties.

6.What do you think is the difference between taking care of dying children and other children?

Taking care of dying children causes psychological and work pressure, I am afraid of mistakes leading to the child's death.

1. What do you think of the impact of this experience on your work or life?

I cherish my life more than before and love my child more than before, I hope my baby can live healthy.

8.Can you describe the most stressful situation you have in taking care of such children? What was the situation?

The biggest pressure is the terrible condition of the child. You know, he was good the day before yesterday, but now he was terrible, the family members could not control the mood blowing, I looked at the lovely child and was repeatedly thinking why. Whether I did not do something well for him？I was also fear of the following problems.

9.Would you please tell me more?

I always feel very anxious with a bad mood. I need a long time to adjust and the experience has affected my work and life.

**Interviewed person: N2**

1. What do you feel when you take care of dying children?

I just wanted to save him, I tried my best, but sometimes the outcome did not change. I could not do anything. I was very depressed.

1. What are your unique feelings about taking care of or rescuing such children?

I feel Helpless, depressed, uncomfortable. Sometimes I cried as I rescued the children. I could not control my own emotions, and I prayed for God to save the children’s life.

1. How do you overcome or relieve this emotion?

Most of the time I would overcome this negative emotion by myself, or speak it out in a group meeting of the department staff, and I would not bring this negative emotion back to my home, nor would I tell my family because I was afraid of affecting the emotions of my family.

1. Would you like to talk how to relieve this stress?

I would buy a bunch of food that I love to eat and find a quiet place. I'll watch some lighthearted and funny TV dramas while eating to distract my attention. Usually I put this pressure in my heart and then slowly forget it. Time will make me forget about bad experiences, and time will make me get used to them.

1. In this case, what do you think is the biggest challenge for you? How do you deal with this challenge? What ways or behaviors or people could help you overcome these challenges?

The biggest challenge is the sense of helplessness, which will permeate all aspects of work and life and affect the normal life. I can not overcome this sense of helplessness and hope that time can make me grow up. At this time, the leadership's recognition of my work is very important to make me realize that I am not too bad.

1. What do you think is the difference between taking care of dying children and other children?

I think the biggest difference is the heaviness of my heart, and caring for a dying child makes me see no hope. If it is to care for a child who is generally not seriously ill, I will be very happy, especially to see the child getting better day by day, I can find personal professional value, but taking care of the dying child, watching him get more and more serious, I am very uncomfortable that I was immersed in sad emotions.

1. What do you think of the impact of this experience on your work or life?

I also have my own baby. I have been working in pediatrics for 12 years. Now I have been trying to change my cognition and accept my inability in this situation. I am trying to face life and work with a positive attitude, and I gradually realize that the health of myself and my family is the most important thing. I will spend more time with their children.

1. Can you describe the most stressful situation you have in taking care of such children? What was the situation?

Generally, if I am the only one in the night shift, I will be under great pressure to rescue the child, because of the workload at night. If there is a situation of rescuing the child, and there is no emergency personnel to be deployed, I will be very flustered, because I can't take care of other children.

9. Would you please tell me more?

In fact, the most important thing is that I hope that professional people can guide this negative mood, especially that the manager can always affirm my working ability, which will be very helpful for me to fight against this mood.

**Interviewed person: N3**

1. What do you feel when you take care of dying children?

I need more time when I care for the dying children, and I want someone to help me, especially when I am on the night shift. Once I was the only one to rescue the child, and other children in the ward were consulting me. I had to save the child and explain to the other family members and let them wait.

1. What are your unique feelings about taking care of or rescuing such children?

I don't know, I'll suffer, I have nightmares. Once I was involved in the rescue of the child, and finally he died. I cooked for him for the body, and I had a nightmare for a month.

1. How do you overcome or relieve this emotion?

I try to avoid this situation at work, and I really can't ask my friends to help me relieve them, and they will tell me their experience and how they deal with it, which I can understand and feel better.

1. Would you like to talk how to relieve this stress?

My friend said that everyone has a way to decompress everyone, some of they listen to music, some to sing, shout loudly to relieve stress. Generally, I can release some pressure, and I usually need the help of others to relieve it.

5.In this case, what do you think is the biggest challenge for you? How do you deal with this challenge? What ways or behaviors or people could help you overcome these challenges?

I think the biggest challenge for me is to question my own ability and the rescue process. I will repeatedly confirm whether the operation process is correct and whether there is any omission. After the rescue, I will constantly recall the rescue process. Did I go wrong throughout the rescue process? Am I not doing it fast enough? Did I missing any details? I can't forgive myself if my problems have failed.

6.What do you think is the difference between taking care of dying children and other children?

The children are so fragile that they are dying. I usually frustrated when I care for them, especially watching them get worse and my care doesn't make them better. Normal children will talk with them and sing, make them laugh, dying children I dare not say loudly.

7.What do you think of the impact of this experience on your work or life?

This experience makes me love my life more. I especially cherish my current work and life. I will take the time to meet with my family and friends and enjoy my life.

1. Can you describe the most stressful situation you have in taking care of such children? What was the situation?

The biggest stress for me should be my unconfidence in my technique, especially if the blood draw or venipuncture is unsuccessful, and I will sweat nervous. I often think that I can get more rescue time if I am highly skilled.

9. Would you please tell me more?

In fact, the affirmation of leaders is crucial to nurses. Once the head nurse told me that you were doing a good job, and I suddenly felt a lot less pressure.

**Interviewed person: N4**

1. What do you feel when you take care of dying children?

I don't feel anything, I am used to it. I can't change the ending. That's the child's life. I also prayed for God, but it was useless, and the child finally died. The parents were very sad, let me not give up, save their children, but I can't change the outcome.

2.What are your unique feelings about taking care of or rescuing such children?

I don't feel much, that's my job. This is what every nurse has to experience, as long as I am still in the nursing profession, so I have no special feeling.

3.How do you overcome or relieve this emotion?

This experience seems to have had little effect on my life, nor would I feel so.

1. In this case, what do you think is the biggest challenge for you? How do you deal with this challenge? What ways or behaviors or people could help you overcome these challenges?

I think the biggest challenge is that nobody helped me, especially when I was on the night shift alone. Usually we have two people on duty in the middle of the night and one in the middle of the night. I was quite afraid that there would be a critically ill child in the ward when I took over in the middle of the night, because it was possible to save him at any time, but I was the only one. I think with one more person on duty with me, I would take my time and be very grateful to my head nurse.

1. What do you think is the difference between taking care of dying children and other children?

It doesn't seem to be much difference to me. I have to engage in nursing work, nothing more than how much energy cost, dying children will spend me a lot of energy, especially the record of nursing documents. The last thing I like is the record of dying children, because I may be involved in a medical dispute.

1. What do you think of the impact of this experience on your work or life?

This experience does nothing particularly affect me because I am used to it. In fact, I don't like nursing work, just because I have studied nursing for several years, and my parents find it a pity not to do this job.

1. Can you describe the most stressful situation you have in taking care of such children? What was the situation?

The biggest pressure for me should be the interference of the family members. The children's parents always stare at me, and I do not dare to make any mistake. I was afraid that they could not accept the death of the child and finally put their anger on my head. Many children's families dare not question the doctor and will only bully the nurses.

8. Would you please tell me more?

As I said before, this is a job my parents chose for me, not the one I like. I just want to work day by day, earn some money and don't be complained about it. I'm actually tired of it, especially every time I experience caring for a dying child.

**Interviewed person: N5**

1. What do you feel when you take care of dying children?

I felt uncomfortable, depressed, my nerves were tight, and I was afraid that the children would die when I was taking care of it.

2.What are your unique feelings about taking care of or rescuing such children?

I am afraid, especially afraid to rescue this kind of children, I have rescued five children, each time I feel not very good, I feel high tension, which leads to my great pressure.

3.How do you overcome or relieve this emotion?

I will stay in a place, eat dessert, cake, drink milk tea. So I don't think back to the bad situation at work.

4. Would you like to talk how to relieve this stress?

In addition to my own ways to alleviate it, I will also ask my colleagues or friends to help me overcome this negative mood. I will tell the whole process every time, and repeatedly tell my friends or colleagues. Colleagues or friends will enlighten me and tell me about their more uncomfortable experience, and I will feel much more happy.

1. In this case, what do you think is the biggest challenge for you? How do you deal with this challenge? What ways or behaviors or people could help you overcome these challenges?

I think the biggest challenge was my technique, when the intravenous needle failed and needed immediate venous access to input the rescue drug, and I tried several times without success. The child's blood vessels were too thin. I had never seen the blood back inside the blood vessel during the puncture process. My nervous hands were full of sweat. Fortunately, a senior nurse helped me, and I think the critical moment of puncture success is too important. I have been trying to improve my technical level, although I still can't overcome the tension every time.

6. What do you think is the difference between taking care of dying children and other children?

When the dying child looked at me for help, I dared not even look back at him, because I was afraid that I would let him down, and I was afraid to see the light in his eyes go out.

7. What do you think of the impact of this experience on your work or life?

This experience makes me change my attitude towards life, and I have become more cherish my life now. Because human life is too fragile, and accidents will happen at any time, I should cherish the people around me in the limited life.

8. Would you please tell me more?

In fact, the first time I had this experience, my feelings were the strongest, and I couldn't accept my inability, and I look forward to a miracle, but there was no miracle. Sometimes life is cruel, we have only to accept its cruelty, and calmly face life. We should learn to use the help of the people around us, and not just rely on ourselves.

**Interviewed person: N6**

1.What do you feel when you take care of dying children?

I don't feel anything, I don't feel anything special, and I think it's my job.

2.What do you think is the biggest challenge in caring for dying children? How do you meet this challenge? What are the ways, behaviors, or people that can help you overcome these challenges?

I think the biggest challenge is to communicate with the families of the child about the child's condition. Because we are a neonatal department, it is a ward without family care, usually nurses are taking care of the children. So once the child's condition changes. Neither of the parents are around, so we need to communicate with our families at this time. But there are some difficulties. The child's parents did not see the child with their own eyes, so the family members could not accept the communication. I think if an older nurse or the head nurse, or the doctor in charge is around me, it can help me overcome this difficult moment.

3. What do you think is the difference between taking care of dying children and other children?

When the child was dying, I even wanted to let him get free. Because of so many tubes inserted in him, the child remained motionless. In this case, I feel particularly uncomfortable. I think since I can not save him, then let him free as soon as possible, and thus reduce his pain.

4.What do you think of the impact of this experience on your work or life?

This experience can affect my work. Although I'm used to it, I still can't accept it. In fact, this experience has always had a potential impact on my life. Although I did not show it on the surface, and my family did not see my change, but actually my heart is still very afraid, so. I have nightmares at night and I will be afraid to go through these.

5. Can you describe the most stressful situation you have in taking care of such children? What was the situation?

I remember once, when I communicated with the child's grandmother about the child, and the child's grandmother asked me to save her grandson again and again. However, whether the child can save the life is not on my opinion, I can not decide the result and can not promise her to save her grandson. So I feel very sick. And I'm also under great pressure.

6. Would you please tell me more?

Because in general, when Chinese parents have only one child, the needs of such family members will be very high, and their expectations will also be very high. Once the child's condition changes, there is no treatment, the child's parents generally can not accept. They will vent all their bad emotions on the nurses.

**Interviewed person: N7**

1.What do you feel when you take care of dying children?

I feel sick and depressed, I don't want to go through these things, I don't want to care for critically ill children. We have a responsibility system for holistic care. I belong to a low seniority nurse, generally responsible for taking care of the children with ordinary conditions, only when they work on the night shift will encounter this situation.

2.What are your unique feelings about taking care of or rescuing such children?

My feeling is mainly uncomfortable, helpless. It's the feeling of being powerless. I really want to change my child's situation, but I can't. I especially hate the feeling.

3.How do you overcome or relieve this emotion?

I am most of the time self-regulating, I will find a quiet corner. Put this experience in the heart, not to recall. Because every time I mention it, I feel very uncomfortable.

4. Would you like to talk how to relieve this stress?

Everyone has a way to relieve the pressure, I usually adjust by myself, using the method is to empty their own brain, not to think about what I have experienced.

5.In this case, what do you think is the biggest challenge for you? How do you deal with this challenge? What ways or behaviors or people could help you overcome these challenges?

I think the biggest challenge is the lack of manpower during the night shift rescue, especially when only one person is on duty at night. At that time, I would feel that obvious that I wish I had three heads and six arms. I would feel much better if anyone helped. Even if my colleagues sleep in the nurse's duty room, I will feel much better.

6. What do you think is the difference between taking care of dying children and other children?

I think the biggest difference is the change of mindset. I knew the child was very sick. I can't joke with him, and I can't tease him. My heart is very heavy, the child's family mood is also very heavy, we basically talk very little communication.

7.What do you think of the impact of this experience on your work or life?

The impact of this experience on my life is that I focus particularly on the health of my own children. Once my child is sick, I love thinking consider the worst. The impact of this experience on my work is that if I encounter this situation frequently for a period of time, I especially want to change my jobs.

8. Can you describe the most stressful situation you have in taking care of such children? What was the situation?

The biggest pressure of taking care of these children is the lack of manpower when on duty at night. Even when I was on the night shift alone, I had a lot of other jobs, and I was too busy to coordinate well. After all, I'm still a junior nurse. So, I, I still hope to work when more people, a little more secure.

9. Would you please tell me more?

In fact, once after I rescued the children, my heart is particularly uncomfortable. It was the head nurse who came over and gave me a hug, and then she told me that I had done very well, and that then I felt a lot less pressure. So I hope that my leadership can often give support and affirmation to my work. After all, I am young, so I particularly want to be recognized by my superiors.

**Interviewed person: N8**

1.What do you feel when you take care of dying children?

So far, I have had two experiences of caring for dying children. My main feeling is depressed, uncomfortable. I felt that my negative emotions were particularly obvious. Every time I take a long time to get down.

2.What are your unique feelings about taking care of or rescuing such children?

I feel myself helpless feeling more obvious. I have no children of my own because I am young. Every time I encounter this situation, I mainly feel that my ability is too poor, there is always a sense of powerless.

3.How do you overcome or relieve this emotion?

I need to overcome this helplessness without constant skill training. I improve my ability by training or watching relevant videos. Although sometimes it may not be because of my own technical problems, but I still think that if I am too strong in my ability, I can get more time, and thus change the outcome of my child.

4.In this case, what do you think is the biggest challenge for you? How do you deal with this challenge? What ways or behaviors or people could help you overcome these challenges?

I think the biggest challenge for me is how to communicate with my family. Whenever the child is seriously ill in need of rescue, the most uncomfortable is the children's families. Because I have no children, I can't really feel the mood of their parents, so sometimes I have no point in the communication with their parents. The parents didn't want to talk to me, either. I think I still need to read some books on communication skills, especially on doctor-patient communication. Especially in this special case or in the critical case of the children, how to communicate effectively with the parents during the rescue time, I think I can exercise my communication skills through situational simulation training.

5. What do you think is the difference between taking care of dying children and other children?

I think the difference is that the care of the dying children themselves is under great pressure. I am afraid that my child's illness will worsen, which will cause the complaints and dissatisfaction of the child's parents.

6.What do you think of the impact of this experience on your work or life?

In fact, this experience is a positive incentive effect for my life. It will constantly urge me to grow up and enable me to constantly strengthen my learning to exercise my technical ability and service level. In the future to better improve their comprehensive ability. I also need to learn from senior nurses.

7. Can you describe the most stressful situation you have in taking care of such children? What was the situation?

I remember once when a child gave up treatment, the head nurse asked me to pull out all the catheter on the child. I felt like I was the most stressed at that time. I looked at the child's parents, and we were all in a bad mood. I didn't know how to communicate to them, and I didn't even know how to speak. The parents were crying all the time, and I did so. So, I think my biggest problem is how to communicate with them when I am very excited at home.

8. Would you please tell me more?

I think the managers should do some relevant training, or I should strengthen the study of relevant knowledge in this aspect. This situational simulation training will help me better cope with this situation.

**Interviewed person: N9**

1.What do you feel when you take care of dying children?

My main mood is the discomfort. I feel very, very uncomfortable. In my life, I am a person with more delicate feelings, especially easily affected by this situation. Usually this kind of thing will affect my mood for a long time.

2.What are your unique feelings about taking care of or rescuing such children?

My feeling was just depressed and uncomfortable. I want to cry, and I want to cry out loud every time I think about it.

3.How do you overcome or relieve this emotion?

I have been working for a long time and have many experiences of caring for dying children. I think that most of the time, I will try to relieve these negative emotions. I might have overcome it a little faster if the care manager could help me ease it. I think nursing managers can organize parties so that people can talk about this experience and feelings.

4. Would you like to talk how to relieve this stress?

Everyone has a way to release pressure, and I usually adjust the negative emotions to try to forget the experience and not think back on it.

5.In this case, what do you think is the biggest challenge for you? How do you deal with this challenge? What ways or behaviors or people could help you overcome these challenges?

The biggest challenge I face is how to communicate effectively with my family members. Although I have experienced it for many times, every time I communicate with my family members, their emotions are different, and every time the situation is different, and I face people with different personalities. I think the head nurse organized the discussion to help me overcome these bad emotions, especially after I experienced such things, if the leader thinks that the ineffective rescue of children is not my ability, it will make me feel much better.

6.What do you think is the difference between taking care of dying children and other children?

Taking care of this kind of child, it may be more stressful. Is that the invisible pressure is very big. Fear is because of their own negligence, resulting in the child suddenly more serious illness, thus rescue invalid.

7. What do you think of the impact of this experience on your work or life?

The experience affects my life is positive. Because after my this experience more, I especially cherish the present life. I want to spend more time with my children. I will also particularly cherish my present work at work.

8.Can you describe the most stressful situation you have in taking care of such children? What was the situation?

In my opinion, the biggest pressure is communication, especially when the family members are very impatient and very excited. Even, sometimes the parents of the children may have physical contact with me and have an accidental injury. Once, the father had just come back from the outside, and he could not accept the change in the child's condition. He thought that I did not do my best to save his child. When he tried to beat me, the doctor stopped him.

9. Would you please tell me more?

Other people I don't think there is anything special to say, because after all, I have learned to be grateful. Sometimes I will have deeper contact and communication with the families of the children, and I also hope that they can get out of this situation as soon as possible.

**Interviewed person: N10**

1.What do you feel when you take care of dying children?

Actually, this job is not a job that I like. The nursing profession was not my own choice, but what my parents helped me choose. So I wasn't very happy either, so if I had this experience, I don't really have any special influence by myself, because I don't have any special feeling originally.

2. In this case, what do you think is the biggest challenge you face? How do you meet this challenge? What are the ways, behaviors, or people that can help you overcome these challenges?

I think the biggest challenge is the small number of nurses, especially when I am on duty alone at night, the high intensity of care will lead to my physical exhaustion, and I have to take care of my children during the day, this situation makes me powerless, I hope someone can help me.

3. What do you think is the difference between taking care of dying children and other children?

For me, there's no difference, that's my job, and what kind of children I care for is all the same for me. And I've worked for years, and I might not have chosen it if it wasn't for my own kids.

4. What do you think of the impact of this experience on your work or life?

This experience may actually have a little more negative impact on my life and work, and I feel like I'm very tired right now. My negative emotions are also becoming more and more, and I often complain about my life. This experience has already affected my work, my life. I don't have a great relationship with my own children either.

5. Can you describe the most stressful situation you have in taking care of such children? What was the situation?

The biggest pressure for me was probably that I was alone on the night shift, and no one helped me. After all, I still have to pick up the children during the day, and I can't have a good rest. The night shift is easily tired. I can't rest during the day.

6. Would you please tell me more?

I think the management personnel should do some relevant training, or I should strengthen the study of the relevant knowledge, which will make me better deal with this situation.
