# Supplementary material for: Experience of pediatric nurses in nursing dying children - a qualitative study
Source: BMC Nurs. 2023 Apr 18;22:126. doi: 10.1186/s12912-023-01274-0 (PMC10111798; doi:10.1186/s12912-023-01274-0)
Supplement: Supplementary file 2 — Supplementary Material 2 [file 12912_2023_1274_MOESM2_ESM.docx]

**Appendix 1 .Search strategies**

| **Date** | 26th January 2020 | |
| --- | --- | --- |
| **Research Topic** | Nurses’ challenges and coping strategy in caring for dying children in China | |
| **Search Strategy** | **Keywords/concepts** | **Synonyms/alternative terminology** |
|  | Challenge | challenges or barriers or difficulties or issues or problems or limitations or obstacles |
|  | Coping strategy | coping strategies or coping skills or coping or cope |
|  | Dying children | Death of a child or pediatric palliative care or dying infant or dying child |
| **Limits and Type of material required** | 2010 -2020 | |
|  | English language | |
|  | Full text | |
| **Databass searched** | Academic Search Complete | |
|  | CINAHL Complete | |
|  | Health Source: Nursing /Academic Edition | |
|  | MEDLINE | |
|  | MEDLINE Complete | |

## Appendix 2: Flow chart


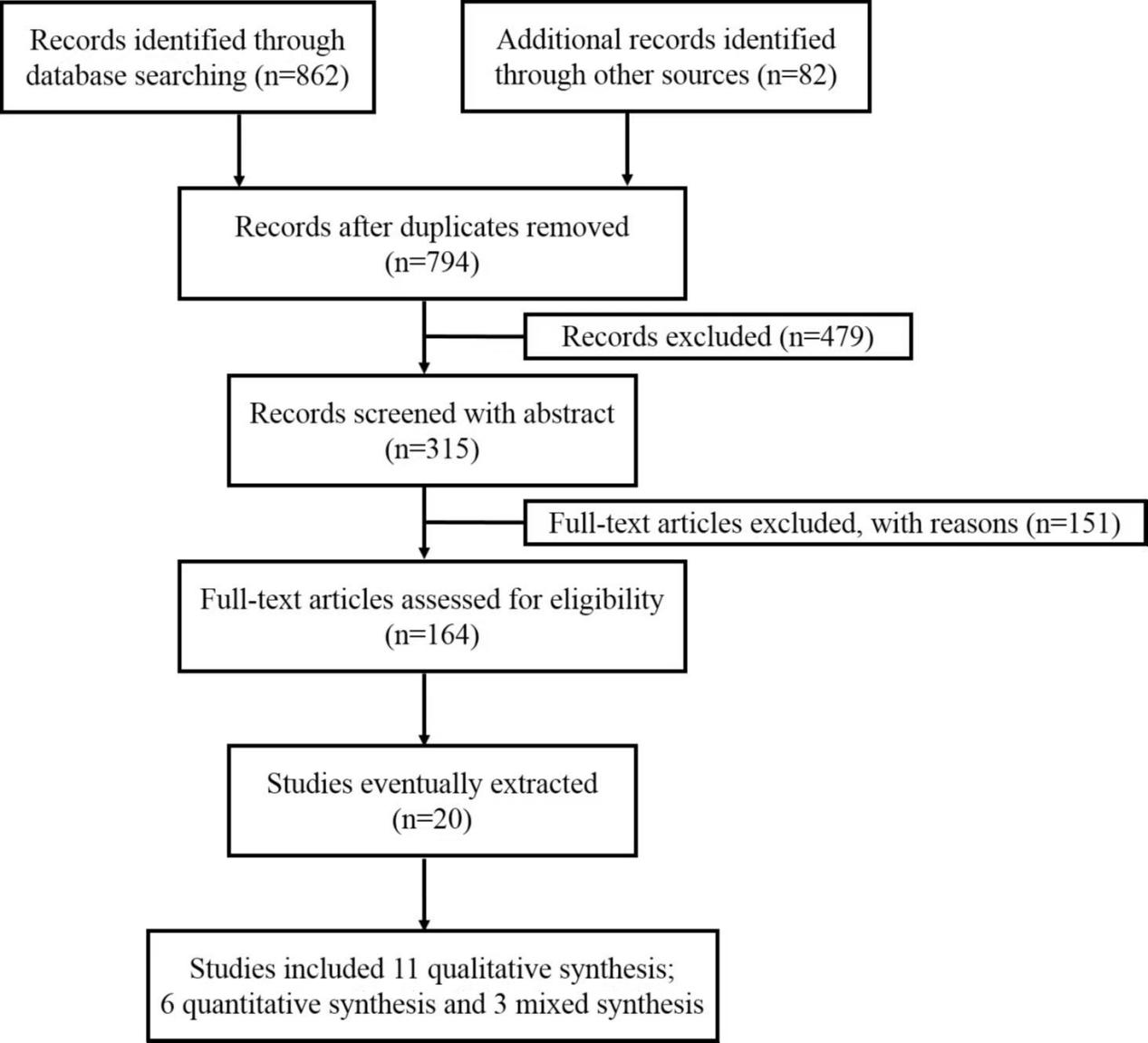


**Appendix 3: The characteristic of participants for Experience Of Pediatric Nurses In Nursing Dying Children - A Qualitative Study**

| participants | gender | Age | Working years | Original department | Marital status | Qualification | Children |
| --- | --- | --- | --- | --- | --- | --- | --- |
| N1. | female | 41 | 21 | pediatric | Married | Junior college | 2 |
| N2. | female | 38 | 12 | pediatric | Married | Junior college | 1 |
| N3. | female | 30 | 8 | pediatric | Married | Undergraduate | 2 |
| N4. | female | 28 | 7 | pediatric | Married | Undergraduate | 1 |
| N5. | female | 49 | 31 | Neonatology | Married | Junior college | 1 |
| N6. | female | 38 | 20 | Neonatology | Married | Junior college | 1 |
| N7. | female | 27 | 6 | Neonatology | Married | Undergraduate | 1 |
| N8. | female | 25 | 3 | Neonatology | Married | Undergraduate | 0 |
| N9. | female | 42 | 22 | Emergency | Married | Junior college | 1 |
| N10. | female | 37 | 25 | Emergency | Divorced | Junior college | 1 |

**Appendix 4. The interview questions for Experience Of Pediatric Nurses In Nursing Dying Children - A Qualitative Study**

| What do you feel when you take care of dying children?  What are your unique feelings about taking care of or rescuing such children?  How do you overcome or relieve this emotion?  Would you like to talk how to relieve this stress?  In this case, what do you think is the biggest challenge for you? How do you deal with this challenge? What ways or behaviors or people could help you overcome these challenges?  What do you think is the difference between taking care of dying children and other children?  What do you think of the impact of this experience on your work or life?  Can you describe the most stressful situation you have in taking care of such children? What was the situation?  Would you please tell me more? |
| --- |

**Appendix 5 Themes and sub-themes extracted from the analysis of the findings**

| Theme | Sub-themes |
| --- | --- |
| Stressors | i) Negative emotions |
|  | ii）Helplessness |
|  | iii) Questioning rescue behavior |
|  | iv）Fear of communication |
|  | v）Lack of workforce for night rescue |
| Stress Consequences | i) Compassion fatigue |
|  | ii) Burnout |
|  | iii) Changes in life attitudes |
| Coping strategies | i) Self-regulation of coping strategies |
|  | ii) Leadership approval and no accountability |
